# Depressive symptoms and associated factor among public school teachers in Jimma town, Southwest, Ethiopia 2020: a multi-disciplinary, cross-sectional study

**DOI:** 10.1186/s12888-022-03941-z

**Published:** 2022-05-18

**Authors:** Tilahun Bete, Kabtamu Gemechu, Tamrat Anbesaw, Hunde Tarafa, Jinenus Tadessa

**Affiliations:** 1 Department of Psychiatry, School of Nursing and Midwifery, College of Health and Medicine Science, Haramaya University, Harar, Ethiopia; 2 Department of Medical Laboratory, College of Health and Medicine Science, Haramaya University, Harar, Ethiopia; 3 Department of Psychiatry, College of Medicine and Health Science, Wollo University, Dessie, Ethiopia; 4 Department of Psychiatry, College of Health and Medicine Science, Mettu University, Mettu, Ethiopia; 5 Department of Psychiatry, College of Health and Medicine Science, University of Gonder, Gonder, Ethiopia

**Keywords:** Depressive symptoms, Prevalence, Magnitude, Teachers, Associated factor, Ethiopia

## Abstract

**Background:**

Depression is a common mental disorder and the leading cause of disability globally. Depression has a significant impact on the quality of life, cognition, emotion, and daily functioning and leads individuals to 39% of suicide globally. Previous studies reported that the magnitude of depression is higher among teachers than in the general population. However, little is known in the case of Ethiopia. Therefore this study aimed to assess the magnitude of depressive symptoms and associated factors among public school teachers in Jimma town.

**Methods:**

A facility-based cross-sectional study using was conducted a multistage random sampling technique. Depressive symptoms were assessed by using the Depression, Anxiety, and Stress 21 items scale. The data were entered into Epi Data version 3.1 and analyzed using STATA V 14.2. Variables with *p* < 0.25 in the bi-variable logistic regression analysis were entered into a multivariable binary logistic regression to identify predictors. A statistically significant association was declared at a *p*-value < 0.05.

**Result:**

The prevalence of depressive symptoms in this study was found to be 44.7% (95% CI: 40–49.3). Moderate and severe level of occupational stress (AOR; 2.63 CI; 1.32, 5.28 and AOR; 4.15 CI; 1.83, 9.45) respectively, having stress, (AOR; 2.40 CI; 1.48, 3.90), having Anxiety symptoms (AOR; 4.43 CI; 2.79, 7.06) and consumption of alcohol (AOR; 2.21 CI; 1.11, 4.37) were identified as a significant predictor for depressive symptoms.

**Conclusion and recommendation:**

The study revealed that there is a high prevalence of depressive symptoms among participants. Moderate and severe levels of occupational stress, having stress, having anxiety, and consumption of alcohol were factors that are significantly associated with depressive symptoms. Therefore, giving awareness and routine screening of depressive symptoms among teachers is crucial in early detection and management.

## Background

Depression is a common cold of mental illness and the leading cause of disability globally [1]. It is characterized by internal feelings of sadness, guiltiness, hopelessness, helplessness, loss of interest in previously pleasurable activities, disturbed sleeping patterns, loss of concentration, loss of appetite, psychomotor retardation or agitation, and suicidal ideation and attempt [2]. According to DSM-5, five symptoms are needed for at least 2 weeks duration and among the symptoms, one must be either depressed mood or loss of interest and cause significant functional impairment [3].

Worldwide 350 million people are suffering from major depression disorder [2, 4]. Out of 28% of neuropsychiatric disorders of the global burden of disease, depression is accountable for more than one-third [5]. It was the third leading cause in 2012 and it was predicted in 2020 to become the second of the burden of global disease [6]. According to World Health Organization estimation; major depressive disorder (MDD) is projected to become the leading by 2030 [7].

Depression is the most known and common disabling problem that leads individuals risky to health like diabetes, arthritis, substance [8] and it also increases the mortality rate by four times than non-depressed [9]. Depression has a significant impact on cognition, emotion, quality of life, and daily functioning [2, 10]. Depression is one of the most common psychiatric disorders that lead individuals to suicide and 39% of global suicide occur by depression [11]. It also has a considerable impact on social disability and economic to both the patient and family.

Depression among teachers varies across the country, in England 19.4 to 52.5% [10, 12–14]. Having 1–3 children, younger age, having genetically predisposing to mental illness, being female, divorced, low income, anxiety, stressful life events, substance use, being diabetes, hypertension, occupational stress, working experience, poor social support were factors that significantly leads to depression [10, 13–19].

Different studies internationally reported that teachers are exposed to major depressive disorder than other professions [17, 20–23]. Teachers are one of the fundamental elements of the education system. They are a role model who motivates and encourages students for great achievement [24]. Teaching is a physically and mentally challenging profession because they waste a lot of energy in their daily occupations in the classroom. In addition to this personal social life and familial commitments leads them to mental distress [25].

The lifetime prevalence of depression among teachers is higher than the general population which ranged between 19.4 to 52.5% among teachers, [10, 12, 14, 19, 23, 26] and 14.6% among the general population [27]. Despite the high magnitude and burden among teachers it is still underestimated public health problem and is well not known in Ethiopia. In addition, this study assesses the role of the level of occupational stress on depressive symptoms among school teachers. This study has paramount significance in early systematic screening and detection of depressive symptoms. It is also an important and effective strategy to prevent further complications from depressive symptoms. Therefore, this study particularly focuses on assessing the magnitude of depressive symptoms and factors associated with depressive symptoms among school teachers in Jimma town, Southwest. Ethiopia.

## Methods and materials

### Study setting and period

The study was conducted from December first to thirty 2020. G.C. at public schools in Jimma town. Jimma town is located in Oromia regional state 352 Km far from the capital city of Ethiopia, Addis Ababa, to the southwest. The estimated total population of the town is 210,908. In the town, there are 33 public schools: among this twenty-seven were primary and six were secondary schools. There are 2018 public school teachers in Jimma town. Among these 1332 are primary school teachers and 686 are secondary school teachers.

### Study design

A facility-based cross-sectional study was employed.

### Source population

All public school teachers working in Jimma town.

### Study population

All randomly selected public school teachers in Jimma town.

### Eligibility criteria

#### Inclusion criteria

All Jimma town public school teachers who are serving for more than 6 months and who are currently in service were included in this study.

#### Exclusion criteria

Teachers who are on maternity and sick leave for different reasons and teachers who are acutely ill during the data collection period were excluded from this study.

### Sample size estimation and sampling technique

#### Sample size estimation

A single population proportion formula was used to estimate the sample size. The prevalence of depression 29.3% among Nigerian teachers was obtained for this objective, at (95% level of confidence), a 5% margin of error. Since the source population is less than 10,000, using correction formula ($\frac{n_o}{1+\frac{n_o}{N}}$) and by considering 1.5 for design effect and 10% non-response rate, the final sample size was 456.

### Sampling technique

**Fig. 1:**
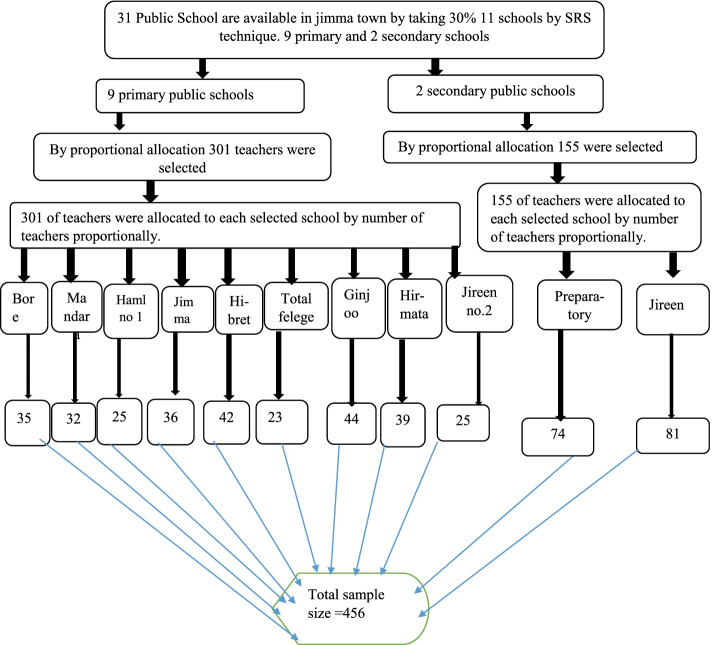
Show proportional allocation of the sample of teachers in the school prepared by the principal investigator in Jimma town Southwest, Ethiopia 2020

A Multi-stage sampling technique was employed. According to the world health organization recommendation first 30% of public schools were selected which means eleven schools from the total of 33 schools by a lottery method of simple random sampling technique. Out of twenty-seven primary schools, nine [9] schools and two secondary schools out of six [6] public schools were included in the study. Then after proportional allocation of the sample size was made based on the number of teachers. From primary schools, 301 teachers are selected out of 1332 teachers, and from secondary school teachers, 155 teachers were selected out of 686 teachers. Finally, a lottery method of simple random sampling technique was used to select study participants from each school teacher by using school teachers’ registration as a sampling frame (Fig. 1).

### Operational definition

**Depressive symptoms:** participants who scored ≥10 in the DASS-21 score were considered as having depressive symptoms.

**Anxiety symptoms**: participants who scored ≥8 in the DASS-21 score were considered as having anxiety symptoms.

**Social support:** Using Oslo Social Support Scale those teachers were scored < 3–8 low social support, score 9–11 (medium social support), and 12–14 (strong social support).

**Level of occupational stress:** Occupational Stress Index (OSI) was used to measure OS. According to the scale total scores, the teachers’ OS was divided into:

- ➢ Mild level of OS (total score: 46–122),
- ➢ Moderate level of OS (total score: 123–155)
- ➢ Severe level of OS (total score:156–230)

**Current substance use:** use of Chat, Alcohol, cigarettes, Cannabis, and other psychoactive substance or medication in the last 3 months.

### Instrument and data collection procedures

A structured interview was employed to collect the data. The first part assesses information about Sociodemographic data. Depression, anxiety, and stress were assessed by adopting the Depression Anxiety Stress Scale 21 (DASS-21) questionnaire. That is a validated and reliable instrument with 21 items in three domains. Each domain comprises seven. Scores from each dimension were added and multiplied by two. Then those who score above between 0 and 9 for depression considered as normal, 10–13 mild depression, 14–20 moderate depression, 21–28 severe depression, and above 28 were considered as an extreme case of depression. For anxiety, those who score above eight and for stress above fifteen were considered as having the case [28–30]. It has excellent Cronbach’s alpha values of 0.81, 0.89, and 0.78 for the subscales of depression. Anxiety and stress respectively [30]. In this study, internal consistency (Cronbach’s alpha coefficient) was 0.82, 0.85, and 0.92 for depression, anxiety, and stress respectively. The level of occupational stress was assessed by using Occupational Stress Index (OSI). The English version of the OSI was originally developed by Srivastava and Singh (1984) and it is applicable in different countries including Africa in Egypt and Algeria among teachers. The scale supports to measure the extent of Occupational stress that employees perceive from various constituents and conditions of their job [31]. It has 46 items scale (28 positives and 18 negatives) that were rated on a 5-point Likert scale ranging from (1: strongly disagree to 5: strongly agree). The items are related to relevant components of job conditions that could be sources of stress as work overload, role ambiguity, role conflict, group and political pressure, responsibility for persons, under participation, powerlessness, poor colleges’ relations, intrinsic impoverishment, and bad working conditions. According to the scale total scores, the teachers’ OS was divided into a mild level of OS (total score range: 46–122), moderate level of OS (total score range: 123–155), and severe level of OS (total score range: 156–230) [31, 32]. The reliability and Cronbach’s alpha coefficient for the scale were 0.935 and 0.90, respectively in Bangladesh [33]. The Oslo 3-items social support scale was used to assess social support. A sum index was made by summarizing the raw scores, the sum ranging from 3 to 14. It was reliable in the study (Cronbach’s α = 0.91) done at Wolayta university [34, 35]. Job burnout was assessed by using the adopted Maslach burnout inventory that has 22 items and has seven scale choices for each question. Those who score above the mean of the Maslach burnout inventory questionnaire were considered as they have Job burnout whereas those who score below the mean were considered as they have not. It has Cronbach’s alpha value of reliability was 0.89 [36]. In this study, internal consistency (Cronbach’s alpha coefficient) was 0.83. current substance user was assessed by using modified ASSIST [37].

### Data quality management

A self-administered questionnaire was employed to collect the data, which was prepared in the local language (Amharic and Afan Oromo). Eleven data collectors from BSc psychiatry professionals and one supervisor from MSc were involved in the data collection process and training was given for 2 days on the data collection tool, content, privacy, consent and confidentiality, and data collection procedure. A pre-test was conducted on 5% of the sample size and adjustments were made based on the pretest.

### Data processing and analysis

The data were entered into the Epi-Data version 3.1, and then data was exported to Stata 14.3. The bivariate logistic analysis was done to select candidate variables. All variables *p*-value < 0.25 in the bivariate analysis were entered into the multivariable logistic regression model. Multivariable logistic regression analysis was employed to control for possible confounding effects and to determine the presence of a statistically significant association between independent variables and outcome variables. The model of fitness was checked by Hosmer and Lemeshow test which was 0.257. A *P*-value < 0.05 was considered statistically significant and the strength of the association was presented by an odds ratio of 95% C.I.

## Result

### Sociodemographic characteristics of respondents

Of the 456 eligible, 452 teachers have participated in the study giving a response rate of 99.12%. More than half 241(53.32%) of participants were female. More than half 237 (52.43%) of the respondents were first-degree holders followed by diploma, masters and certificate 126(27.88), 52 (11.50%), 37 (8.19) respectively. and most of the respondents were married followed by single, divorced, separated, and widowed 313(53.3%), 54(11.95%), 34(7.52%), 32(7.08%), and 19(4.20%) respectively. The mean age of the respondents was 41.71 with a standard deviation of (+ 10.91). Regarding teachers’ monthly salary most of the respondents paid more than five thousand five hundred Ethiopian birrs 294(65.04%) and 94(20.80%) of the paid less than 4500 Ethiopian birrs, the remaining were paid in between.

### Work and mental health characteristics of participants

Three fourth of the participant 298 (65.93%) were primary school teachers and 247(54.65%) of them teach social science. Among all participants, most of them 297 (65.71%) were suffered from moderate occupational stress followed by severe occupational stress 82(18.14%) and the remaining have a mild level of occupational stress. and half of the participants 226 (50%) experienced job burnout above the mean level. More than half of the respondents 267 (59.07%) have teaching experience above 15 year 115(25.44%) of them have teaching experience between 6 and 15 years and the remaining have less than 5 years of teaching experience. Among the total respondents, 202 (44.69%) of them have anxiety symptoms and 78 (17.26%) of them have a family history of mental illness.

### Substance and medical illness characteristics of respondents

Among the respondents, 51(11.28%) and 30 (6.64%) were current alcohol and cigarette smokers respectively. Regarding medical illness, 34 (8.52%) diabetic Mellitus, 37 (8.19%) hypertension, and 28 (6.19%) asthma.

### Prevalence of depression among respondents

**Fig. 2:**
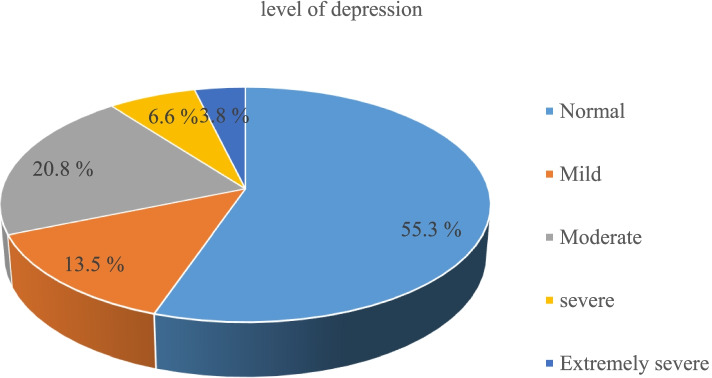
Level of depression among public school teachers in Jimma town, Southwest Ethiopia, 2020

The total prevalence of depression among respondents is 202 (44.7%) (95% CI: 40–49.3) (Fig. 2).

### Factors associated with depression among teachers in Bi-variable logistic regression

Bi-variable logistic analysis was done to see factors associated with depression; Hence, marital status, level of the school, family history of mental illness, current alcohol user and smoker, social support, level of occupational stress, anxiety, perceived stress, Job burnout, having diabetic Mellitus and hypertension were found to be associated with depression and entered to multivariate analysis (Table 1).

**Table 1:** Factors associated with depression in Bi-variable logistic regression among respondents in Jimma town in 2020 (*N* = 452)

Variable	Characteristics	Depression		COR	CI	***P***-value
		Yes	No			
Sex	Male	94	117	Ref	Ref	Ref
	Female	108	133	1.01	0.69, 1.47	0.955
Age	21–30	38	43	0.99	0.57, 1.70	0.964
	31–40	53	80	0.74	0.46, 1.19	0.217
	41–50	43	51	0.94	0.56, 1.59	0.823
	> 50	68	76	Ref	Ref	Ref
Educational	Certificate	18	19	1.26	0.55, 3.01	0.554
	Diploma	60	66	1.24	0.65, 2.38	0.518
	Degree	102	135	1.03	0.56, 1.89	0.923
	Masters	22	30	Ref	Ref	Ref
Marital status	Married	132	181	Ref	Ref	Ref
	Single	30	24	1.71	0.96, 3.06	0.069
	Divorced	11	23	0.66	0.31, 1.39	0.272
	Separated	18	14	1.76	0.85, 3.67	0.130
	Widowed	11	8	1.89	0.74, 4.816	0.185
Level of School	Primary school	145	153	Ref	Ref	Ref
	Secondary school	57	97	0.62	0.42, 0.92	0.019
Subject taught	Social science	106	141	Ref	Ref	Ref
	Natural Science	96	109	1.17	0.81, 1.70	0.405
Teaching experience	≤ 5 year	35	35	1.22	0.72, 2.07	0.450
	6–15 year	47	68	0.85	0.54, 1.32	0.462
	≥15 year	120	147	Ref	Ref	Ref
Salary	< 4500 Ethiopian Birr	42	52	1.03	0.65, 1.648	0.891
	4500–5500 Ethiopian Birr	31	33	1.2	0.69, 2.065	0.506
	> 5500 Ethiopian Birr	129	165	Ref	Ref	Ref
Student behavior	Not feel disturbed	85	98	Ref	Ref	Ref
	Feels disturbed	115	148	0.89	0.61, 1.31	0.570
FHMI	Have no FHMI	168	206	Ref	Ref	Ref
	Have FHMI	34	44	0.95	0.58, 1.55	0.830
Current Alcohol User	Not alcohol user	172	229	Ref	Ref	Ref
	Alcohol User	30	21	1.90	1.05, 3.44	0.033
Current Smoker	Not Smoker	30	21	Ref	Ref	Ref
	Smoker	18	12	1.94	0 .91, 4.13	0.085
Level of occupational stress	Mild OS	18	55	Ref	Ref	Ref
	Moderate OS	132	165	2.44	1.37, 4.36	0.002
	Severe OS	52	30	5.29	2.64, 10.63	0.000
Social support	Poor Social Support	81	75	1.76	1.07, 2.88	0.025
	Moderate Social Support	78	105	1.21	0.75, 1.95	0.437
	Strong Social Support	43	70	Ref	Ref	Ref
Stress	Have no Stress	65	172	Ref	Ref	Ref
	Have Stress	137	78	4.65	3.12, 6.92	0.000
Anxiety	Have no Anxiety	63	187	Ref	Ref	Ref
	Have Anxiety	139	63	6.55	4.34, 9.89	0.000
Job burnout	Not experienced burnout	93	133	Ref	Ref	Ref
	experienced burnout	109	117	1.33	0.92, 1.93	0.130
Diabetic Mellitus	No Diabetic Mellitus	180	234	Ref	Ref	Ref
	Have Diabetic Mellitus	22	16	1.79	0.91, 3.50	0.091
Hypertension	Have No Hypertension	181	234	Ref	Ref	Ref
	Have Hypertension	21	16	1.69	0.86, 3.34	0.127

### Factors associated with depression among teachers in multivariable logistic regression

In multivariable logistic regression analysis of current alcohol users, levels of occupational stress, anxiety, and perceived stress were found to be significantly associated with depression (Table 2).

**Table 2:** Factors associated with depression among teachers in multivariable logistic regression in Jimma town in 2020(*N* = 452)

Variable	Characteristics	Depression		COR	AOR	CI	***P***-value
		Yes	No				
Marital status	Married	132	181	Ref	Ref	Ref	Ref
	Single	30	24	1.71	1.51	0.75, 3.01	0.247
	Divorced	11	23	0.66	0.53	0.21, 1.32	0.175
	Separated	18	14	1.76	1.48	0.59, 3.72	0.386
	Widowed	11	8	1.89	1.95	0.58, 6.52	0.279
Level of School	Primary school	145	153	Ref	Ref	Ref	Ref
	Secondary school	57	97	0.62	0.77	0.48, 1.26	0.300
FHMI	Have no FHMI	168	206	Ref	Ref	Ref	Ref
	Have FHMI	34	44		0.82	0.45, 1.49	0.506
Current Alcohol User	Not alcohol user	172	229	Ref	Ref	Ref	Ref
	Alcohol User	30	21		2.21	1.11, 4.37	0.023
Current Smoker	Not Smoker	30	21	Ref	Ref	Ref	Ref
	Smoker	18	12		0.68	0.25, 1.85	0.447
Level of occupational stress	Mild OS	18	55	Ref	Ref	Ref	Ref
	Moderate OS	132	165		2.63	1.32, 5.28	0.006
	Severe OS	52	30		4.15	1.83, 9.45	0.001
Social support	Poor	81	75		1.19	0.65, 2.18	0.575
	Moderate	78	105		0.06	0.59, 1.89	0.834
	Strong	43	70	Ref	Ref	Ref	Ref
Stress	Have no Stress	65	172	Ref	Ref	Ref	Ref
	Have Stress	137	78		2.40	1.48, 3.90	0.000
Anxiety	Have no Anxiety	63	187	Ref	Ref	Ref	Ref
	Have Anxiety	139	63		4.43	2.79, 7.06	0.000
Job burnout	Not experienced burnout	93	133	Ref	Ref	Ref	Ref
	experienced burnout	109	117		0.96	0.60, 1.53	0.875
Diabetic Mellitus	No Diabetic Mellitus	180	234	Ref	Ref	Ref	Ref
	Have Diabetic Mellitus	22	16		1.46	0. 62, 3.46	0.386
Hypertension	Have No Hypertension	181	234	Ref	Ref	Ref	Ref
	Have Hypertension	21	16		1.36	0. 57, 3.22	0.485

*Ref* Reference, *COR* Crudss Odds Ratio, *AOR* Adjusted odds ratio, *CI* Confidence interval

## Discussion

This study aimed to assess the prevalence and associated factors of depressive symptoms among teachers living in Jimma town. Overall the study revealed that 44.7% (95% CI: 40–49.3) of teachers are suffering from depressive symptoms. Among this 13.3, 20.8, 6.6, 3.8% are mild. Moderate, severe, and extremely severe respectively. Regarding associated factor level of occupational stress, having anxiety, perceived stress and current alcohol user were significantly associated with depressive symptoms.

The prevalence of depressive symptoms in the current study is in line with the finding of Libya 44.5% [38] and Malesia 43% [19]. But it is lower than the study conducted in India 52.5% [39]. Sample size variation, lifestyle condition, the socio-cultural background might be another possible reason, tool difference is also another difference. In India, depression was assessed by the Beck Depression Inventory (BDI) [39], while in our study Depression, Anxiety, and Stress Scale (DASS-21) was used.

The finding of this study is higher than the study in England 19.4% [10], Italy 23.9%) Japan 20.1% [26] Egypt, 23.2% [12], Nigeria 29.3% [13]. The possible explanation for the difference might be tool differences used to assess depression. They used Patient Health Questionnaire (PHQ-9), England Beck Depression Inventory (BDI), Egypt Zung’s Self-Rating Depression Scale (SDS) japan, MINI International Neuropsychiatric Interview Nigeria to assess depression respectively but in this study, Depression Anxiety Stress Scale (DASS-21) item was used. The pandemic effect of COVID-19, Sample size variation, lifestyle condition, socio-cultural background difference might be another possible reason.

The odds of having depression is 2.65 and 4.15 more likely to occur among having a moderate and severe level of occupational stress respectively than having a mild level of occupational stress AOR = 2.63 at 95% CI (1.32, 5.28), and AOR = 4.15 at 95% CI (1.83, 9.45) respectively. This agrees with the study done in Japan [26], in Mexico [14]. The possible reason for this might be job demands may exceed individuals coping abilities that might predispose them to develop depressive symptoms through psychological (like the feeling of helplessness) [23].

Teachers who report current alcohol users were more than two times more likely two have depressive symptoms than not current user AOR = 2.21 at 95% CI (1.08, 4.26). This is supported by the study conducted in Mexico [14]. The possible explanation might be the effect of alcohol consumption on the brain it decreases the serotonin concentration in the blood which is responsible the predisposing them to depressive symptoms [2, 40, 41]. The other reason might be individuals who consume alcohol might develop depression symptoms like loss of concentration, social isolation, and feels depression.

Teachers who have perceived stress were more than two times increased risk of having depressive symptoms than teachers who have no perceived stress AOR = 2.40 at 95% CI (1.48, 3.90). This is consistent with studies done in England, Brazil, and Malaysia [42–44]. This is might be stress harms mental health because individuals’ degree of perception for events whether it is stress full or not and loss of feeling that the situation is out of their control to increase the individual’s risk of developing depressive illness [45]. Likewise, teachers having anxiety increases the likelihood of having depression by 4.43 times as compared to teachers who do not have anxiety AOR =4.43 95% CI (2.79, 7.06). The reason for this might be anxiety most commonly comorbid with depression [2].

## Conclusion and recommendation

The study revealed that there is a high prevalence of depression among participants. Moderate and severe levels of occupational stress, having stress, having anxiety, and consumption of alcohol were factors that are significantly associated with depression. Therefore, giving awareness through education about depression and routine screening of depression among teachers is crucial in early detection and management. Furthermore, for researchers, it is better to use both qualitative and quantitative study designs for further understanding of depression.

## Data Availability

The datasets used and/or analyzed during the current study are available from the corresponding author on reasonable request.

## Abbreviations

AOR: Adjusted Odd Ratio
COR: Crude Odd Ratio
DASS: Depression, Anxiety and stress scale
DM: Diabetic Mellitus
GOVT: Government
HTN: Hypertension
KM: Kilometer
MDD: Major Depressive Disorder
NGO: Non-Governmental Organization
OR: Odds Ratio
OS: Occupational stress
OSI: Occupational Stress Index
PI: Principal Investigator
USA: United States of America
WHO: World Health Organization

## References

[1] Herrman H, Kieling C, McGorry P, Horton R, Sargent J, Patel V (2019). Reducing the global burden of depression: a lancet–world psychiatric association commission. Lancet.

[2] Kaplan &Sadock BJ. Synopsis of Psychiatry Behavioral Sciences/Clinical Psychiatry. 2014. 1495–1450.

[3] American Psychiatric Associationorders. Diagnostic and Statistical Manual of Mental Disorders fifth edition (DSM-5). 2013. 1–695.

[4] Federici S, Bracalenti M, Meloni F, Luciano JV (2017). World Health Organization disability assessment schedule 2.0: An international systematic review. Disabil Rehabil.

[5] Mental health report: new understanding, new hope. World Heal Organ. 2001;1–9.

[6] Health TFM of, Ethiopia. National Mental Health Strategy of Ethiopia. 1–19.

[7] World Health Organization. Global Health risks: mortality and burden of disease attributable to selected major risks [internet]. Bull World Health Organ. 2009;87:646–6 Available from: http://www.who.int/healthinfo/global_burden_disease/GlobalHealthRisks_report_full.pdf.

[8] Alemu H, Tsui A, Ahmed S, SA. (2012). Effect of depressive symptoms and social support on weight and CD4 count increase at HIV clinic in Ethiopia. AIDS Care.

[9] M. M. Depression: a global crisis. World Federation for Mental Health. 20th Anniversary of World Mental Health Day. 2012.

[10] Kidger J, Brockman R, Tilling K, Campbell R, Ford T, Araya R (2016). Teachers’ wellbeing and depressive symptoms, and associated risk factors: A large cross-sectional study in English secondary schools. J Affect Disord.

[11] WHO. Preventing suicide: a global imperative. Geneva: World Health Organization; 2014 (http://apps.who.int/iris/bitstream/10665/131056/1/9789241564779_eng.pdf?. accessed 7. Geneva World Heal Organ. 2017;1.

[12] Desouky D, Allam H (2017). Occupational stress, anxiety and depression among Egyptian teachers. J Epidemiol Glob Health.

[13] Asa FT, Lasebikan VO (2016). Mental Health of Teachers: Teachers ’ Stress , Anxiety and Depression among Secondary Schools in Nigeria. Int Neuropsychiatr Dis J.

[14] Soria-Saucedo R, Lopez-Ridaura R, Lajous M, Wirtz VJ (2018). The prevalence and correlates of severe depression in a cohort of Mexican teachers. J Affect Disord.

[15] Patten SB, Williams JVA, Lavorato DH, Wang JL, McDonald K, Bulloch AGM (2015). Descriptive epidemiology of major depressive disorder in Canada in 2012. Can J Psychiatr.

[16] Wonder M, Birkie M, Getinet W (2020). Magnitude of depression and associated factors among high and preparatory public school teachers in Gondar town, Northwest, Ethiopia 2020.

[17] Eaton WW, Antony JC, Mandel W, Roberto G (1990). Occupations and prevalence of major depressive disorder. J Occup Med.

[18] Gaias LM, Gal DE, Abry T, Taylor M, Granger KL (2018). Diversity exposure in preschool: longitudinal implications for cross-race friendships and racial bias. J Appl Dev Psychol.

[19] Besse R, Howard K, Gonzalez S, Howard J (2015). Major depressive disorder and public school teachers: evaluating occupational and Health predictors and outcomes. J Appl Biobehav Res.

[20] Johnson S, Cooper C, Cartwright S, Donald I, Taylor P, Millet C. The experience of work-related stress across occupations. 2005;20(2):178–87. 10.1108/02683940510579803.

[21] Stansfeld SA, Rasul FR, Head J, Singleton N (2011). Occupation and mental health in a national UK survey. Soc Psychiatry Psychiatr Epidemiol.

[22] Wieclaw J, Agerbo E, Mortensen PB, Bonde JP, Scandinavian S, Wieclaw J, et al. Occupational risk of affective and stress-related disorders in the Danish workforce published by : the Scandinavian journal of work, Environment & Health, the Finnish Institute of Occupational Health, the Danish National Research Centre for the Working. 2005;31(5):343–51. 10.5271/sjweh.917 16273960

[23] Melchior M, Caspi A, Milne BJ, Danese A, Poulton R, Moffitt TE (2007). Work stress precipitates depression and anxiety in young, working women and men. Psychol Med.

[24] Yilmaz F, Ilhan M (2017). Who are teachers ? A study of identity. Cogent Educ.

[25] Ahmad J (2017). Occupational stress among school Teachers : a review. Int J Innov Res Adv Stud.

[26] Nakada A, Iwasaki S, Kanchika M, Nakao T, Deguchi Y, Konishi A (2016). Relationship between depressive symptoms and perceived individual-level occupational stress among Japanese schoolteachers. Ind Health.

[27] Bromet E, Andrade LH, Hwang I, Sampson NA, Alonso J, de Girolamo G (2011). Cross-national epidemiology of DSM-IV major depressive episode. BMC Med.

[28] Lovibond PF (1995). Pergamon the structure of negative emotional states : scales ( DASS ) with the beck depression and. Elsevier Sci Ltd.

[29] Coker AO, Coker OS, DO. (2018). Psychometric properties of the 21-item depression anxiety stress scale (DASS-21). African Res Rev.

[30] Tran TD, Tran T, Fisher J (2013). Validation of the depression anxiety stress scales ( DASS ) 21 as a screening instrument for depression and anxiety in a rural community-based cohort of northern Vietnamese women. BMC Psychiatry.

[31] Latif A, Sultana S (2009). Adaptation of an occupational stress index. J Life Earth Sci.

[32] Lim CT (2013). The scale of occupational stress in the business process outsourcing industry. Int J Sci Appl Inf Technol.

[33] Jeyaraj SS (2013). Occupational stress among the teachers of the higher secondary schools in Madurai District, Tamil Nadu. IOSR J Bus Manag.

[34] Abiola T, Udofia O, Zakari M (2013). Psychometric properties of the 3-item Oslo social support scale among clinical students of Bayero University Kano, Nigeria. Malaysian J Psychiatry.

[35] Kocalevent RD, Berg L, Beutel ME, Hinz A, Zenger M, Härter M (2018). Social support in the general population: standardization of the Oslo social support scale (OSSS-3). BMC Psychol.

[36] Maslach C, Jackson SE. The Measurement of Experienced Burnout Author ( s ): Christina Maslach and Susan E. Jackson Published by : Wiley Stable URL : https://www.jstor.org/stable/3000281 The measurement of experienced burnout *. J Occup Behav 1981;2(2):99–113.

[37] Humeniuk RE, Henry-Edwards S, Ali RLPV, MM. (2010). The alcohol, smoking and substance involvement screening test (ASSIST): manual for use in primary care.

[38] Taher YA, Samud AM, Hashemi MM, Kabuoli NF (2016). Prevalence of depression, anxiety and stress among Libyan primary and secondary Schoolteachers : a cross-sectional study. Jordan J Pharm Sci.

[39] Shetageri VN GG. A Cross-Sectional Study Of Depression And Stress Levels Among School Teachers Of Bangalore. Available from: www.iosrjournals.org. IOSR J Dent Med Sci Ver VII. 2016;15(3):2279–861.

[40] Swendsen JD, Merikangas KR, Canino GJ, Kessler RC, Rubio-Stipec M, Angst J (1998). The comorbidity of alcoholism with anxiety and depressive disorders in four geographic communities. Compr Psychiatry.

[41] Manninen L, Poikolainen K, Vartianen E, Laatikainen T (2006). Heavy drinking occasions and depressions. Alcohol Alcohol.

[42] Othman Z, Sivasubramaniam V (2019). Depression, anxiety, and stress among secondary school teachers in Klang, Malaysia. Int Med J.

[43] Mendes Rodrigues LT, Lago EC, Landim Almeida CAP, Ribeiro IP, Mesquita GV (2020). Stress and depression in teachers from a public education institution. Enferm Glob.

[44] Mahan PL, Mahan MP, Park NJ, Shelton C, Brown KC, Weaver MT (2010). Work environment stressors, social support, anxiety, and depression among secondary school teachers. AARON J.

[45] Kinser PA, Lyon DE. A conceptual framework of stress vulnerability, depression, and health outcomes in women: potential uses in research on complementary therapies for depression. Brain Behav. 2014;4:665–74. 10.1002/brb3.249 PMC4107381 25328843

